# High N Storage but Low N Recovery After Long-Term N-Fertilization in a Subtropical *Cunninghamia lanceolata* Plantation Ecosystem: A 14-Year Case Study

**DOI:** 10.3389/fpls.2022.914176

**Published:** 2022-06-15

**Authors:** Fangfang Shen, Wenfei Liu, Honglang Duan, Jianping Wu, Chunsheng Wu, Yingchun Liao, Yinghong Yuan, Houbao Fan

**Affiliations:** ^1^Jiangxi Provincial Key Laboratory for Restoration of Degraded Ecosystems and Watershed Ecohydrology, Nanchang Institute of Technology, Nanchang, China; ^2^Jiangxi Provincial Key Laboratory of Soil Erosion and Prevention, Jiangxi Academy of Water Science and Engineering, Nanchang, China; ^3^Institute for Forest Resources and Environment of Guizhou, Key Laboratory of Forest Cultivation in Plateau Mountain of Guizhou Province, College of Forestry, Guizhou University, Guiyang, China; ^4^Yunnan Key Laboratory of Plant Reproductive Adaptation and Evolutionary Ecology, Yunnan University, Kunming, China; ^5^Key Laboratory of Soil Ecology and Health in Universities of Yunnan Province, School of Ecology and Environmental Sciences, Yunnan University, Kunming, China

**Keywords:** long-term N fertilization, N storage, plant organs, ecosystem components, *Cunninghamia lanceolata* plantation

## Abstract

Forests are among the most important N pools of all terrestrial ecosystems. Elevated atmospheric N deposition in recent decades has led to increased interest in the influences of N application on forest N cycles. However, accurate assessments of N storage in forest ecosystems remain elusive. We used a 14-year experiment of a Chinese fir [*Cunninghamia lanceolata* (Lamb.) Hook] plantation to explore how long-term N fertilization affected N storage and recovery rates. Our study plots were located in a field that had been continuously fertilized over 14 years (2004–2017) with urea at rates of 0 (N0, control), 60 (N60, low-N), 120 (N120, medium-N), and 240 (N240, high-N) kg N hm^−2^a^−1^. Data were collected that included N content and biomass in the understory, litter, and various plant organs (i.e., leaves, branches, stems, roots, and bark), as well as soil N content and density at different depths. Results showed that the total ecosystem N storage in the N-fertilized plots was 1.1–1.4 times higher than that in the control plots. About 12.36% of the total ecosystem N was stored in vegetation (plant organs, litter, and understory) and 87.64% was stored in soil (0–60 cm). Plant organs, litter, and soil had higher N storage than the understory layer. Significantly higher plant N uptake was found in the medium-N (1.2 times) and high-N (1.4 times) treatments relative to the control. The N recovery rate of the understory layer in the N-fertilized treatments was negative and less than that in the control. Application of long-term N fertilizer to this stand led to a low N recovery rate (average 11.39%) and high loss of N (average 91.86%), which indicate low N use efficiency in the Chinese fir plantation ecosystem. Our findings further clarify the distribution of N in an important terrestrial ecosystem and improve our understanding of regional N cycles.

## Introduction

Nitrogen (N) is a fundamental constituent of many biomolecules, including proteins and nucleic acids, and therefore one of the most limiting factors on plant growth and productivity (Nadelhoffer, 2008; Xu et al., 2020; Qubain et al., 2021). N concentration in terrestrial ecosystems is particularly sensitive to anthropogenic disturbance, including N fertilization (Bouwman et al., 2002; Tian et al., 2006; Etzold et al., 2020; Verma and Sagar, 2020). Unfortunately, human activity has led to ubiquitous excessive and inappropriate application of N fertilizers that may influence the global N cycle (Lu et al., 2011), upsetting the balance of N uptake among forest plants (Liu et al., 2020 and references therein), alter forest ecosystem structure and function, and inhibit ecosystem productivity by tipping nutrient balances (Nadelhoffer, 2008; Tian et al., 2018; Li et al., 2019). N fertilization also reduces ecosystem biodiversity through soil acidification and ammonium toxification (Talhelm et al., 2013; Zhang et al., 2014; Midolo et al., 2019; Wu et al., 2021), and can decrease ecosystem stability *via* altered dominant species abundance and plant functional group composition (Wu et al., 2020).

Forests are the largest N pools of terrestrial ecosystems and account for 35.86% of the total N storage in China (Xu et al., 2020). Changes in forest N pools caused by N application can alter global N cycle processes (Fenn et al., 2020; Xu and He, 2020; Zhang et al., 2020). “Closed” N cycles are formed in the soil-litter-plant continuum found in forest ecosystems, which are sensitive to N deposition (Nadelhoffer, 2008; Liu and Wang, 2021; Qubain et al., 2021). In this closed N cycle, annual plant N uptake per unit area is balanced by annual N return to litter and soil (Nadelhoffer, 2008). Several studies have demonstrated that the mass balance of N in a forest ecosystem is generally characterized by the difference between N inputs, vegetation and soil N sinks, N leaching, and gaseous loss (Lovett and Goodale, 2011; Gurmesa et al., 2016). In forest ecosystems, external N inputs, especially soil fertilizers, are first applied to the soil and then absorbed and used by plants (Qubain et al., 2021). Most N is retained by litter and soil, and only a small fraction is taken up and retained by plants (Nordin et al., 2001; Gurmesa et al., 2016; Wang et al., 2018; Li et al., 2019). The N uptake capacity of soils and vegetation may be a major factor determining tree growth and mortality in response to N addition (Wallace et al., 2007; Lovett and Goodale, 2011). The N recovery rate is often used as an indicator of N use efficiency (Ju and Zhang, 2003; Tian et al., 2011; Congreves et al., 2021). Even small fluctuations in the forest N pool can dramatically influence global N cycles (Xu and He, 2020). For example, external fluctuations of N inputs decreased main ecosystem compartments recovery to different degree in “N-rich” tropical forest (Gurmesa et al., 2016). Mapping the distribution of fertilized N in forest ecosystem components therefore represents the importance of N storage and N recovery to forest ecosystems N cycles.

Although numerous studies have focused on how N fertilization affects forest ecosystems, it is widely concerned that the response of ecosystem N cycle to N fertilization various in short-term and long-term N application, because the responses of forest ecosystems to N fertilization cannot completely reveal in a short time (Zhu et al., 2015). Previous studies have been reported that N application tended to have positive effect on litter N pool and soil N cycle processes (i.e., accelerating soil N mineralization rate) in short-term fertilization experiment (Lu et al., 2011; Zhu et al., 2015), but had negative effects when forest reached N saturation in long-term N fertilization experiments, such as reduced soil microbial biomass and respiration (Zhu et al., 2015; Zhang T. A. et al., 2018). However, N-induced changes in the aboveground plant N pool did not show any significant correlations with experimental duration (Lu et al., 2011). The effects of fertilized N depend greatly on the fate of fertilized N (Gurmesa et al., 2016); thus, empirical studies that follow N over long time scales are critically needed to better understand how N fertilization influences ecosystem N cycle.

It is widely accepted that excessive N fertilization enhances the leaching of ecosystem nutrients (Wilson and Tiley, 1998), especially in N-saturated forests (Gurmesa et al., 2016). In a review of studies across China, Tian et al. (2018) found that an N application rate exceeding 80 kg N hm^−2^a^−1^ can be regarded as an extremely high level of N in forests. Further, rates higher than 90 kg N hm^−2^a^−1^ can lead to negative feedbacks between soil N availability and transformation (Verma and Sagar, 2020). High levels of N input can cause forest decline (Wilson and Tiley, 1998). For example, one study found that N fertilization at 150 kg N hm^−2^a^−1^ over 15 years induced high tree mortality (Högberg et al., 2006). Two common symptoms of N-induced damage are nutrient deficiencies caused by accelerated plant growth and a loss of soil base cations and species by soil acidification (Högberg et al., 2006; Zhang Y. Q. et al., 2018). N leaching loss can range from 2% to 20%, with higher N dosages leading to higher losses (Tian et al., 2018). While several studies have addressed the effects of N fertilization on plant growth and nutrient cycles in forest ecosystems over short time spans, they provided limited information on the long-term effects of high N fertilization on forest N uptake and N storage, particularly with respect to N recovery rate.

The average rate of N deposition in China has increased by approximately 60% over the past three decades (Yu et al., 2019), up to 150 kg N hm^−2^a^−1^ in some areas (Zhang et al., 2014). Higher leaf, aboveground plant and litter N pools were observed when N application rate from 0–50 to 50–100 kg N hm^−2^a^−1^, but no more increase from 50–100 to 100–150 kg N hm^−2^a^−1^ (Lu et al., 2011). While rapid plant species loss in long-term low frequency of N application showed the same results with the high rates (Zhang et al., 2014). The rate of N application in this study is much higher than the atmospheric N deposition rate (33.2 kg N hm^−2^a^−1^ on average) across this region (Xu et al., 2018). In previous work we showed that our current study site is N-saturated (Wu et al., 2017, 2021); thus, an extremely high dose of N fertilizers (i.e., 120 and 240 kg N hm^−2^a^−1^) provides a unique opportunity for studying the effects of high N inputs on forest ecosystems.

Forests cover about 220 million ha in China, which is 22.96% of the global terrestrial ecosystem (FAO, 2015; FRA, 2020). China’s plantation forests are the largest in the world, accounting for 27% of the total global area of planted forests (FAO, 2015). Chinese fir [*Cunninghamia lanceolata* (Lamb.) Hook] is an economically important species in China (Zhang et al., 2020), covers an area of 8.93 million ha (Nation Forestry and Grassland Adminstration, 2019). *Cunninghamia lanceolata* does not fix N_2_ and therefore depends upon combined or fixed forms of mineral N (Tian et al., 2018). The objective of our study was to accurately estimate the N storage of *C. lanceolata* plantation ecosystem after 14 years N fertilization. Since 2004, we carried out an N deposition experiment in subtropical region with four urea-N fertilization rates (0, 60, 120, and 240 kg N hm^−2^a^−1^), with the aim to assess the allocation of N amongst plant organs (i.e., leaves, branches, bark, stems, and roots) and ecosystem components (plants, understory, litter, and soil), as well as N recovery rate. We hypothesized that: (1) N storage of both *C. lanceolata* and the total ecosystem would increase with fertilization rate under long-term N fertilization and (2) N recovery rate would decrease with N fertilization rate.

## Materials and Methods

### Study Site

Our experiments site is located in Guanzhuang National Forest Farm (117°43′E, 26°30’N), Sanming City, Fujian Province in southeastern China, at an altitude of approximately 200 m. This area has a mid-subtropical monsoon climate with abundant rainfall, characterized by an average annual temperature of 20.1 ± 1.96°C, precipitation of 2,777 ± 40.2 mm a^−1^ (>80% of which falls from May–October, Supplementary Figure 1), and a frost-free period of 271 days (climatology based on measurements from 2004 to 2017).

The Chinese fir plantation was planted in 1992 over an area of 6 hm^2^ at a density of 1,660 individual trees per hectare. There were no legumes in this area, and it had received no fertilizer prior to this experiment. In December 2003, 12 plots (20 m × 20 m each and with a 15 m × 15 m central area) were randomly selected within the forest with a minimum distance between plots of 10 m. The average tree height of the entire plantation was 12 m, and the mean diameter at breast height (DBH, 1.3 m from the ground) was 16.1 cm. A background investigation conducted in 2003 revealed that the understory was sparse, with coverage between 3% and 5%; dominant understory species included *Miscanthus floridulus*, *Dicranopteris olichotoma*, and *Pteridium aquilinum* var. *Latiusculum* (Wu et al., 2013; Shen et al., 2019a). We also measured soil physical and chemical properties; the soil was acidic (pH = 4.67) with an organic carbon content of 18.39 g kg^−1^, a total N content of 0.79 g kg^−1^, and was classified as an Acrisol (Fan et al., 2014; Shen et al., 2019a; Wu et al., 2021).

### Experimental Design

This N fertilization experiment started on January 1, 2004. There were four rates of ureas-N fertilization, 0, 60, 120, and 240 kg N hm^−2^a^−1^, referred to as N0 (control), N60 (low-N), N120 (medium-N), and N240 (high-N), respectively. Each treatment was applied to three replicate plots on the same mountain slope. The N-plots were fertilized monthly and continued to 2017 [14 years prior to Shen et al. (2019a)]. According to the N concentration of each treatment, urea fertilizer was weighed and then dissolved in 20 L of water; and the solution was sprinkled evenly with a backpack sprayer to the forest floor of each plot. The control plots received the equivalent 20 L of water alone.

### Field Sampling

Sampling was conducted on 15 December 2017. Following a per-wood inspection, DBH statistical analysis over the treatment period (2004–2017), and the map of all Chinese firs in plots, we selected a standard tree (normal growth, no pests or diseases, and few scars) in each plot for wood tests, for a total of 12 standard trees subjected to biomass measurements. We determined the orientation of the selected trees with a compass. Before felling, we marked the orientation of the stem at breast height. The central cross-sectional differentiation quadrature method was used to intercept the stem disc (5 cm thick) at the midpoint of each zone segment (every 2 m of the whole tree beginning at the coarse roots). After felling, plant organs (i.e., leaves, branches, stems, bark, and roots) samples were collected from various points and locations in the field per standard tree per plot and weighed. Briefly, leaves were taken from various direction and composite to one sample; branch, bark, and stem samples were taken from various points and locations. The root system biomass was measured following total root excavation (Niiyama et al., 2010). Root samples were divided into coarse roots (>10 mm) and fine roots (<2, 2–5, and 5–10 mm) and were cleaned with deionized water and weighed. Then all subsamples (5 kg of each fresh mass) were transported to the laboratory.

Forest litter was sampled using a 1 m × 1 m frame (three random samples per plot); the samples were separated into an undecomposed layer (L layer) and a semidecomposed layer (F layer), and the layers were then evenly mixed into composite samples, in total, 12 plots × 2 layers × 3 replicate = 72 composite litter samples. The L layer was distinguished by litter with leaves still maintaining their original shape, color, and without superficial evidence of decomposition; by contrast, the leaves of the F layer were crushed with degraded outlines and mostly decomposed mesophyll. All litter samples were brought back to the laboratory to measure water content, total N content.

Six soil cores, 2.5 cm in diameter, down to 60 cm and divided the core into 0–20, 20–40, and 40–60 cm soil sample, and thoroughly composited the six cores from each plot to end up with three replicate soil samples from each depth, in total, 12 plots × 3 depths × 3 replicate = 108 composite soil samples, for soil water content and total N content analysis. The soil samples were transported to the laboratory, sieved through a 2-mm mesh to remove nonsoil materials, and then divided into two sub-samples. One sub-sample was oven-dried at 105°C until the weight remains constant for soil water content determination. The other sub-sample was air-dried then powdered to sieve through a 0.15-mm mesh for analysis of soil total N content. We took an additional 5 cm inner diameter core at each of the three replicate plots from the 0–20, 20–40, and 40–60 cm depths, taking care to collect the exact volume the core, for soil bulk density determination.

To capture the understory species diversity when more seasonal vegetation was visible, we investigated the diversity of the understory on 16 September 2017. Three 5 m × 5 m survey subplots were randomly set in each plot, and we recorded the identity, height, and crown width of species, as well as the number of small trees, lianas, herbs, and shrubs taller than 5 cm in each plot. The biomass of the understory layer was determined by harvesting; small trees and shrubs were collected from 2 m × 2 m sampling areas, and herbs were collected from 1 m × 1 m sampling areas. Whole understory plants were excavated and divided into above- and belowground components. The aboveground components consisted of leaves, branches, and stems of small trees, and leaves and branches of herbs, while the belowground components consisted of roots.

Stem disc samples were taken at breast height and air-dried in ventilated conditions. They were sanded with sandpaper until the annual rings were visible. The discs were then cross-dated to the tree core samples at each sample point and tree-ring width was determined using LinTab 6 (TSAP-Win, Germany).

### Laboratory Analysis

After being returned to the laboratory, all fresh mass of each sample was measured, followed by determine water content, dry biomass, and N content. All fresh leaves were first washed with 10% dilute hydrochloric acid and then repeatedly washed with deionized water to completely remove dust and particulates adsorbed on the surface, then heat-killed at 105°C for 15 min and oven-dried at for 48 h (Liu and Wang, 2021). The branch and fine root samples were washed with clean water, heat-killed at 105°C for 15 min, and then dried at 75°C for 48 h to a constant weight (Liu and Wang, 2021). The bark and litter samples were dried at 85°C to a constant mass. The dry biomass of plant organs was calculated per unit area based on the water content. All vegetation subsamples (i.e., plant organs, understory, and litter samples) were crushed with a microplant crusher, passed through a 100-mesh sieve, and kept in a dry environment prior to analysis. The total N content of plant organs, litter, understory samples was determined through initial digestion with H_2_SO_4_–H_2_O_2_ followed by measurement with the micro-Kjeldahl method (Liu and Wang, 2021). The soil N content was determined by the Kjeldahl method after digestion with H_2_SO_4_ (Shen et al., 2019a). Soil bulk density cores were sieved to 4 mm and oven-dried (105°C for 48 h; Soong et al., 2020), and bulk density was estimated as the mass of the oven-dried soil divided by its volume.

### Calculation

#### Basal Area Increment

We calculated the basal area increment (BAI, cm^2^) using the following equation:


(1) $$BAI=\pi \;\left(R_{n}{}^{2}–R_{n-1}{}^{2}\right)$$

where *n* is the number of tree rings and R*_n_* is the radius of the *n*^th^ ring (cm).

#### Soil N Storage

Soil N density (*N_d_*, kg m^−2^) refers to the storage of N in the soil layer at a specific depth per unit area and was calculated using the following equation:


(2) $$N_{di}=0.1\times T_{Ni}\times \gamma \times H_{i}\times \left(1-\frac{\delta }{100}\right)$$

where 0.1 is the conversion factor, *i* represents the soil layer (cm), *T_Ni_* represents the total soil N content in soil layer *i* (%), *γ* represents the soil bulk density (g cm^−3^), *H* represents the soil depth (cm), and *δ* indicates the percentage of gravel in soil with a diameter>2 mm (%).

Soil N storage (*N_S,_* t hm^−2^) was calculated as:


(3) $$N_{s}=\sum \left(N_{\mathrm{di}}\times A\right)$$

where *A* represents area (hm^−2^).

#### Plant N Uptake and Recovery Rate

We used the definition of fertilizer N recovery efficiency *(RE_N_)* as the percentage of fertilizer N that is taken up by the plant, accounting for background soil N levels (Congreves et al., 2021). The definition is also sometimes referred to as the apparent recovery (Tian et al., 2011; Congreves et al., 2021), calculated as:


(4) $$PlantN_{f}=Plant_{Biomass}\times N\%$$

(5) $$RE_{N}=\frac{PlantN_{f}-PlantN_{0}}{FertilizerN}\times 100\%$$

where *N* (%) is the N content of major ecosystem components, *Plant_Biomass_* (t hm^−2^) is the plant biomass of a plot in this study, *PlantN_f_* (kg N hm^−2^) is the total plant N uptake measured in above- plus belowground biomass in a plot that received N dose (*Fertilizer N*; i.e., 0, 60, 120, or 240 kg N hm^−2^a^−1^), and *PlantN_0_* is the total plant N uptake in unfertilized N plots (N0).

### Statistical Analysis

We used two-way ANOVA with a *post hoc* Tukey test to detect the effects of N treatments and components (litter components, understory layers, and ecosystem components) on biomass, N content, and N uptake; the effect of N treatments and soil layers on density, N content, and N storage; and the effect of N treatments and DBH class on number of trees. One-way ANOVA with Dunnett’s *post hoc* LSD test was used to determine the effects of the N treatments on plant biomass, N content, N uptake, understory species, soil N content, and N storage. All values were given as means ± standard error (*SE*). Results were considered significant at *p* < 0.05. All analyses were conducted using SPSS 19.0 (SPSS, Inc., Chicago, IL, United States). Figures were created with SigmaPlot 13.0 (Sysat software Inc., San Jose, CA, United States).

## Results

### Variation in Soil N Storage

**Figure 1:**
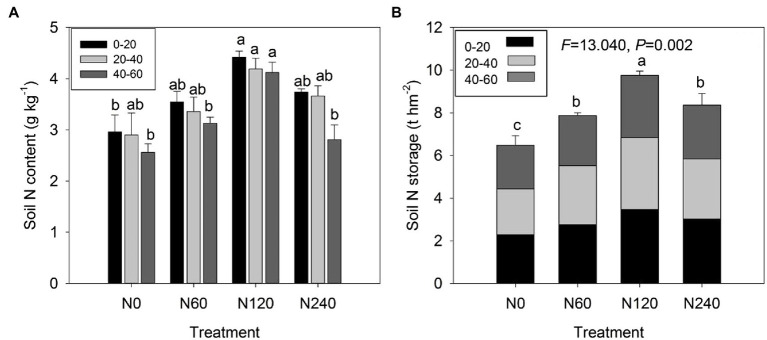
Soil N content **(A)** and N storage in total **(B)** at three soil depths (0–20, 20–40, and 40–60 cm) under different N treatments. N0, control; N60, low-N; N120, medium-N; and N240, high-N. The values show means ± SE (*n* = 3). Lowercase letters indicate significant differences at *p* < 0.05 between different treatments.

N fertilization significantly increased soil N content and storage (*F* = 13.040, *p* = 0.002), which was maximal under the N120 treatment (Figure 1). There were significant effects of N fertilization on the N content of all three soil layers: 0–20 cm (*p* = 0.018), 20–40 cm (*p* = 0.048), and 40–60 cm (*p* = 0.003; Table 1; Figure 1A). Soil N storage showed significant differences in all three layers among the four N treatments [*p* = 0.024 (0–20 cm), *p* = 0.044 (20–40 cm), *p* = 0.014 (40–60 cm); Table 1; Figure 1B]. The N120 treatment increased soil N storage in the 0–20 cm layer by 32.53% compared to that in the N treatment but did not have an effect in the 20–40 or 40–60 cm layers.

**Table 1:** Two-way ANOVA of soil density, N content, and N storage in three soil layers under different N treatments.

Variables	*F* (*P*) value					
	Density (kg m^−2^)		N content (g kg^−1^)		N storage (t hm^−2^)	
Treatment	2.898	0.056	18.711	**<0.001**	5.392	**0.027**
Layer	11.948	**<0.001**	4.99	**0.015**	12.00	**<0.001**
Treatment × layer	0.877	0.526	0.603	0.725	0.877	0.527

Values in bold are statistically significant at *p* < 0.05.

### Variation in DBH and BAI

**Figure 2:**
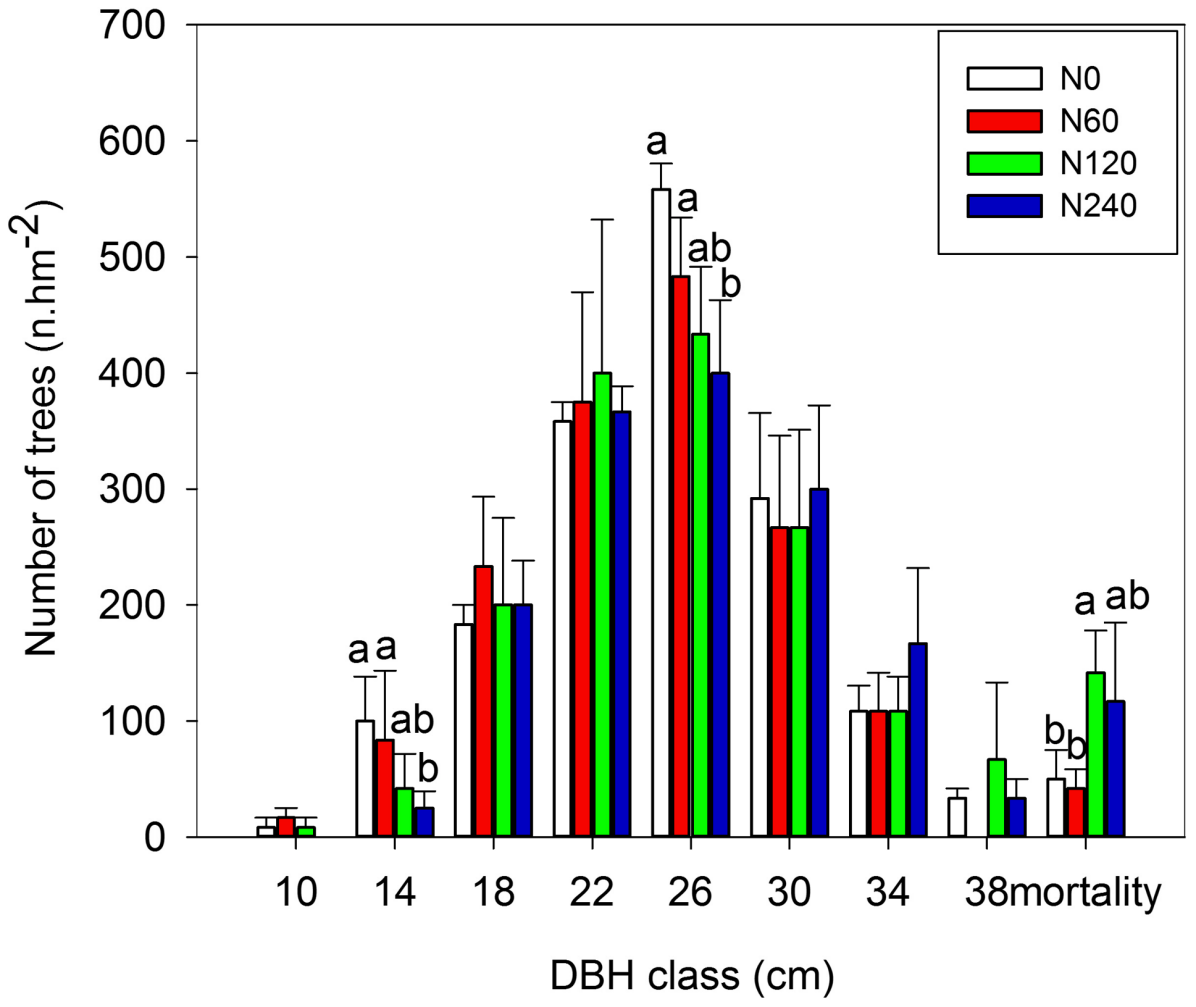
Distribution of trees among DBH classes after 14-year N treatments. DBH classes were defined in 4 cm intervals from 10 cm to 38 cm (in total nine classes); dead trees are all placed in a tenth class as “mortality.” There were no trees with diameters less than 10 cm in the N240 treatment. Within each class, the values of *F* and *p* are shown based on one-way ANOVA. N0, control; N60, low-N; N120, medium-N; and N240, high-N. The values show means ± SE (*n* = 3). Lowercase letters indicate significant differences at *p* < 0.05 between different treatments.

The tree diameters were normally distributed (Figure 2), but there was no significant difference in average DBH among N treatments (Supplementary Figure 2). High-N fertilizer significantly decreased the number of trees with a DBH between 10 and 14 cm, but increased the number of trees with a DBH > 30 cm (Figure 2). Tree mortality was significantly lower in the N0 (2.98%) and N60 (2.55%) treatments compared to the N120 (8.76%) and N240 (7.32%) treatments, with medium-N significantly increased mortality. During the N treatment period (2004–2017), significant differences in BAI and annual BAI between N treatments occurred only in 2013 (*p* = 0.032) and 2014 (*p* = 0.031; Supplementary Figure 3).

### Variation in Vegetation N Storage

**Figure 3:**
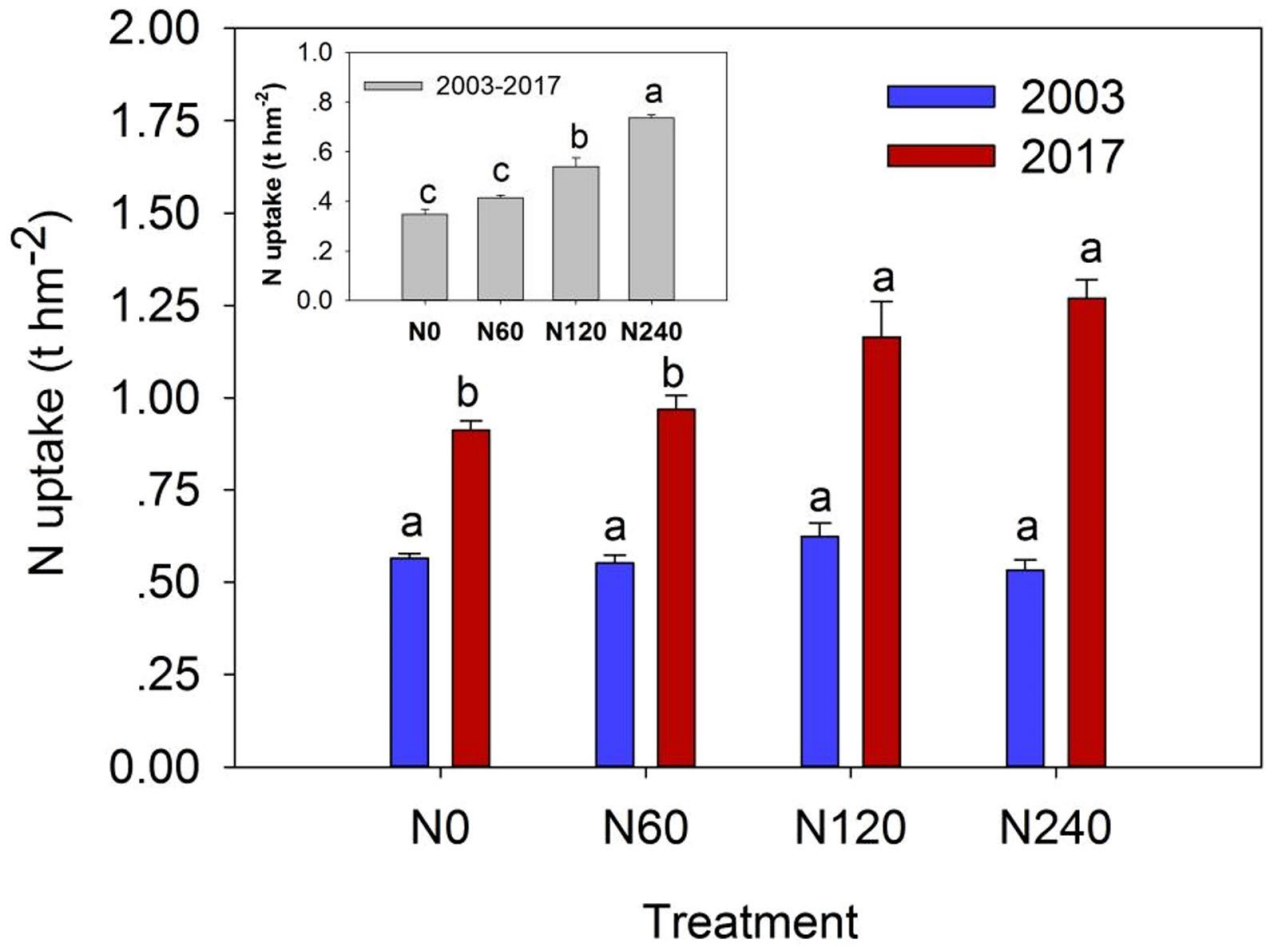
Plant N uptake under different N fertilization rates in 2003 (blue bars), 2017 (red bars), and their differences from 2003 to 2017 (gray bars). Plant N uptake means the sum of N uptake in plant organs (i.e., leaves, branches, bark, stems, and roots). N0, control; N60, low-N; N120, medium-N; and N240, high-N. The values show means ± SE (*n* = 3). Lowercase letters indicate significant differences at *p* < 0.05.

The dry mass and N content of each plant organ were very similar but nonsignificant differences across N treatments (Table 2). N content of leaves and fine roots showed approximately two to four times higher than other organs. Total N uptake of plant organs in the N120 and N240 treatments increased by 27.61% and 39.16%, respectively, relative to that in N0. Significant differences were detected in total plant N uptake in 2017 (*F* = 7.906, *p* = 0.009) and over the study period from 2003 to 2017 (*F* = 60.257, *p* < 0.001) between N treatments (Figure 3; Supplementary Table 1).

**Table 2:** Dry mass and N content of Chinese fir plant organs under different N fertilizer treatments in 2017.

Component	Dry mass (t hm^−2^)				N content (g kg^−1^)			
	N0	N60	N120	N240	N0	N60	N120	N240
Leaves	7.55(0.14)	7.36(0.13)	7.67(0.14)	7.82(0.14)	12.81(1.30)	13.31(1.03)	14.23(1.65)	14.99(1.07)
Branches	19.13(0.50)	18.46(0.47)	19.58(0.52)	20.10(0.51)	6.94(0.31)	7.11(0.43)	7.73(0.54)	7.97(1.18)
Stems	115.25(4.01)	109.71(3.67)	118.90(4.23)	122.79(4.12)	3.00(0.35)	3.48(0.40)	3.78(0.46)	4.86(0.49)
Bark	16.87(0.45)	19.48(0.24)	22.91(0.78)	18.55(0.36)	7.62(0.12)	7.59(0.23)	8.01(0.18)	8.18(0.07)
Coarse roots	35.77(4.82)	34.17(2.41)	42.62(11.61)	33.60(3.37)	4.97(0.15)	5.29(0.21)	5.76(0.27)	6.41(0.59)
**Fine roots**								
<2 mm	0.57(0.07)	0.53(0.22)	0.32(0.10)	0.37(0.05)	13.87(0.48)	13.82(0.86)	14.55(1.54)	15.36(1.28)
2–5 mm	0.97(0.23)	0.86(0.28)	0.65(0.13)	0.62(0.22)	12.88(0.37)	12.65(0.48)	12.47(1.42)	13.48(0.73)
5–10 mm	0.79(0.16)	0.67(0.14)	0.66(0.35)	0.86(0.18)	11.98(0.43)	12.87(0.49)	13.66(1.24)	14.44(1.47)
Subtotal	196.87	191.24	213.31	204.71				

Values are means ± SEs (*n* = 3).

**Figure 4:**
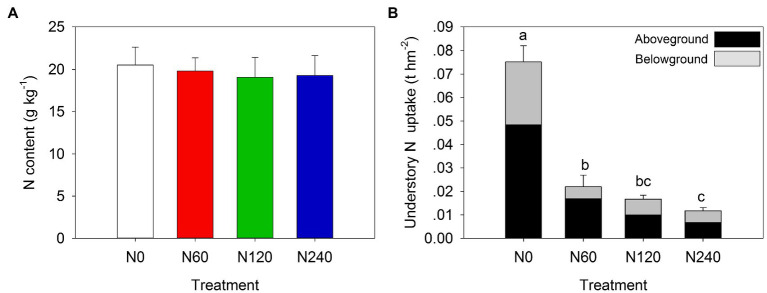
Changes in understory layer N content **(A)** and N uptake **(B)** under different N treatments. N0, control; N60, low-N; N120, medium-N; N240, high-N. The values show means ± SE (*n* = 3). Lowercase letters indicate significant differences at *p* < 0.05 between different treatments.

The understory diversity decreased with increasing N fertilization rates, with species counts of 50, 33, 28, and 16 in N0, N60, N120, and N240, respectively (Supplementary Figure 4A). The abundance of small trees and lianas decreased sharply (approximately 4-fold) in N120 and N240 (*P*<0.05) compared to that in N0. Similarly, the total, above- and belowground understory biomass in the N-fertilized treatments were significantly lower than those in control (*p* < 0.05; Supplementary Figure 4B). Understory layer N content showed no difference (Figure 4A), but N uptake significantly decreased with increasing N fertilization rates (*p* < 0.05) and aboveground N uptake contributed an average of 64.69% of the total understory N uptake (Figure 4B).

**Figure 5:**
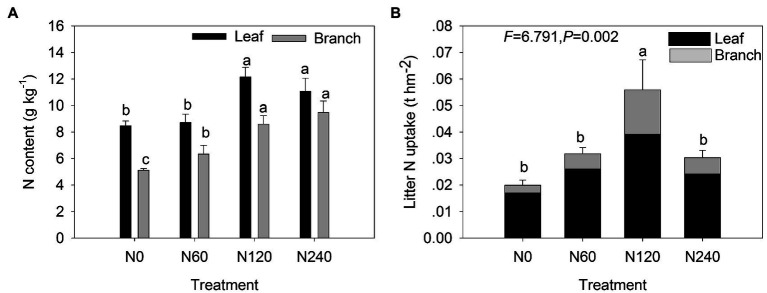
Total litter N content **(A)** and N uptake in total, leaves, and branches **(B)** under different N fertilization treatments. N0, control; N60, low-N; N120, medium-N; and N240, high-N. The values show means ± SE (*n* = 3). Lowercase letters indicate significant differences at *p* < 0.05 between different treatments.

Significant differences were detected in total litter biomass among N treatments (Figure 5; Table 3). The total litter biomass in N60 (5.23 t hm^−2^) was higher than that in N0 (2.55 t hm^−2^; 104.81% increase; *p* < 0.05; Supplementary Figure 5A). The biomass of F-leaf and F-branch in N60 was 1.5 and 4.5 times higher, respectively, than that in N0 (Supplementary Figure 5B). N fertilization had significant effects on the N content on both leaf litter (*F* = 15.577, *p* < 0.001) and branch litter (*F* = 25.026, *p* < 0.001), especially in the N120 treatment (Figure 5A; Table 3). The greatest increase in N uptake was observed in N120 for the total litter and litter components (Figure 5B; Table 3). Leaf litter contributed more to total litter N uptake (up to 79.41%) than branch litter. The branch N uptake in N120 was more than 4.5 times higher than that in N0.

**Table 3:** Two-way ANOVA of biomass, N content, and N uptake under different N treatments and in different litter components.

Variables	*F* (*P*) value					
	Biomass (t m^−2^)		N content (g kg^−1^)		N uptake (t hm^−2^)	
Treatment	5.367	**0.003**	14.865	**<0.001**	10.928	**<0.001**
Component	39.727	**<0.001**	32.267	**<0.001**	66.213	**<0.001**
Treatment × component	0.505	0.681	0.903	0.461	0.590	0.625

Component: leaves and branches in litter. Treatment: N fertilizer treatments, i.e., N0, N60, N120, and N240.

Values in bold are statistically significant at *p* < 0.05.

### Variation in Ecosystem N Recovery Rate

**Figure 6:**
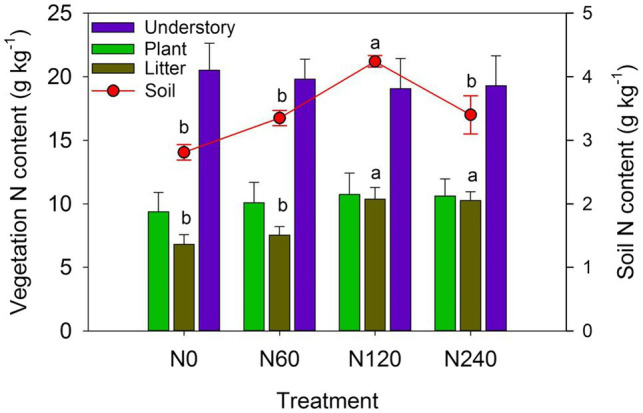
N content of major components of the *Cunninghamia lanceolata* plantation ecosystem under different N fertilizer treatments. Plant: the sum of leaf, branch, stem, bark, fine root, and coarse roots of *C. lanceolata*. N0, control; N60, low-N; N120, medium-N; and N240, high-N. The values show means ± SE (*n* = 3). Lowercase letters indicate significant differences at *p* < 0.05 between different treatments.

Among the major components of *C. lanceolata* ecosystem, only the N content of litter and soil differed significantly between N treatments (Figure 6). N fertilization increased total ecosystem N storage (Table 4), with average N storage of 12.10% in vegetation and 87.9% in soil in the N treatments and 13.46% in vegetation and 86.54% in soil in the control.

**Table 4:** N storage distribution in the Chinese fir plantation ecosystem under different fertilization treatments (t N hm^−2^).

N fertilizer rate (kg N hm^−2^a^−1^)	Chinese fir plant	Litter	Understory layer	Soil	Total in ecosystem
N0	0.91 ± 0.03	0.0199 ± 0.0019	0.0752 ± 0.0068	6.47 ± 0.46	7.48 ± 0.26
N60	0.97 ± 0.04	0.0318 ± 0.0024	0.0219 ± 0.0050	7.87 ± 0.15	8.90 ± 0.10
N120	1.17 ± 0.10	0.0559 ± 0.0113	0.0167 ± 0.0017	9.75 ± 0.20	10.99 ± 0.15
N240	1.27 ± 0.06	0.0303 ± 0.0028	0.0117 ± 0.0014	8.36 ± 0.54	9.67 ± 0.35

Chinese fir plant: leaves, branches, stems, bark, fine roots, and coarse roots of Chinese fir. Litter: litter leaves and branches in both L and F layers. Understory layer: small trees, lianas, shrubs, and herbs. Soil: total N storage of three soil layers (0–20 cm, 20–40 cm, and 40–60 cm). Total in ecosystem: the sum of Chinese fir plant, litter, understory layer, and soil. N0, control; N60, low-N; N120, medium-N; N240, high-N.

The plant N recovery rate in N120 was much higher than that in N60 and N240; the N recovery rates in vegetation were 2.41%, 19.11%, and 12.67% in N60, N120, and N240, respectively, relative to the control; the N recovery rates of the understory layers in the N fertilizer treatments were lower than in the control (Table 5). The N recovery rates of the plant and litter were all positive among N fertilizer treatments, and the N recovery rate was highest in N120 (20.98% in plants and 3.00% in litter). The N storage of vegetation was 14.44, 229.27, and 304.07 kg N hm^−2^ higher in N60, N120, and N240, respectively, relative to N0 (Supplementary Table 2). When N storage in vegetation was compared with the total amount of N fertilizer from 2004 to 2017, the loss of N was estimated to be 825.56 (98.28%), 1450.73 (86.25%), and 3055.9 (90.95%) kg hm^−2^ in N60, N120, and N240, respectively (Supplementary Table 2). Together, our results suggest that long-term N fertilization might induce low ecosystem N use efficiency.

**Table 5:** Recovery rate of N (%) by vegetation from N fertilizer of the 60, 120, and 240 kg hm^−2^a^−1^ treatments relative to the control.

N fertilizer rate (kg N hm^−2^a^−1^)	Chinese fir plant	Litter	Understory layer	Vegetation
N60	9.31	1.98	−8.88	2.41
N120	20.98	3.00	−4.88	19.11
N240	14.88	0.43	−2.65	12.67

Vegetation: the sum of all plant organs, litter, and understory layer.

## Discussion

### Tree Growth and Mortality

Although tree mortality rate in the studied forest (2004–2017) was generally low (average 6.21%), it is noteworthy that tree mortality was higher in the treatments with N fertilization. This is consistent with findings of significantly increased mortality after 18 years of N deposition (30 kg N hm^−2^a^−1^; Talhelm et al., 2013). Tree mortality was sensitive to N deposition, especially at high levels of nitrate deposition, which led to increased tree mortality due to N saturation (Midolo et al., 2019) and soil acidification (Dietze and Moorcroft, 2011; Lovett and Goodale, 2011). Some researchers have attributed this phenomenon to a decrease in the Ca:Al and Mg:N ratios in the foliage and organic soil layer (Aber et al., 1998; Wallace et al., 2007; Lovett and Goodale, 2011). In addition, Fenn et al. (2020) identified a small but significant effect whereby N deposition increased mortality due to crown damage, and Tian et al. (2018) proposed that this increase in tree mortality may indirectly relate to increases in plant disease, changes in soil microbial communities, and greater susceptibility to insect and fungal pathogens. Our finding of increased tree mortality suggests that high N fertilization rates negatively affect trees.

Uniquely, we observed increases in BAI and annual BAI after 10–11 years N treatment, which confirms that N fertilization leads to an increase in relative tree growth (Wallace et al., 2007). The co-occurrence of increased mortality and growth in surviving trees is considered a “threshold” phenomenon in N-saturation forest. The inherent susceptibility of the study site to nitrate leaching and soil acidification in response to N addition, which is mainly regulated by soil and vegetation N uptake capacity, may be the main factor determining tree growth and mortality (Wallace et al., 2007). Enhanced tree growth by N fertilization in this study is inconsistent with results reported by Tian et al. (2018) and Midolo et al. (2019). It has been reported that elevated N deposition had minor effect on forest growth in N-rich forest ecosystems through enhancing transpiration and maintaining nutrient balance (Lu et al., 2018).

### N Storage in the Ecosystem

The N contents differed significantly in the soil and litter between N treatments, but did not vary in plant organs or understory. The neutral response to N fertilization on plant organs might be *C. lanceolata* is a non-N_2_ fixing species (Tian et al., 2018 and references therein). We observed significant increases in soil N content with N fertilization up to a threshold (N120, 120 kg N hm^−2^a^−1^), above which no further increase was observed. The soil N content in N120 was increased by 19.24%–51.49% compared with the control after N fertilization, while results of a meta-analysis by Tian et al. (2018) suggested an increase of soil N content in subtropical (5.4%) and up to 26.9% in tropical forests.

As expected, we found consistently negative effects of high N fertilization rates on understory layer species diversity, biomass, N uptake, and N recovery rate. This supports findings that plant total N uptake controls species dominance (Tian et al., 2018) and that high levels of N fertilization decrease understory layer diversity (Gilliam et al., 2019). Results of Wu et al. (2021) at the same study site found that, over 13 years N addition, understory plant communities significantly decreased. N addition has previously been reported to reduce understory layer biomass and result in the replacement of dominant understory species, which Talhelm et al. (2013) and Midolo et al. (2019) attributed primarily to different N sensitivities and tolerances.

The control plots in our study provide us with clear insights into the growth and N storage of *C. lanceolata* under natural N deposition. Under no added N fertilizer, growth of *C. lanceolata* over 14 years resulted in substantial N storage (7.48 t N hm^−2^), although N fertilization greatly promoted plant N storage. This effect was greatest in the N120 and N240 treatments, which had about 1.47x and 1.30x the N storage of the control treatment, respectively (Figure 3; Table 3). However, the highest N application was not associated with the highest ecosystem N storage, which only partially supports our first hypothesis. The positive effects of N fertilization on the forest ecosystem could be largely driven by the comprehensive effects of N cycles. However, the negative effects on N storage can result from extremely high N fertilization rates. High soil N storage in forest ecosystems and changes in labile soil N factors in response to extremely high N application rates are undeniable (Tian et al., 2011, 2018).

### Higher N Storage After 14 Years of N Fertilization

N fertilizer applied to ecosystem can follow three major routes: absorption by plants, remain in the soil, or loss through several pathways (Ju and Zhang, 2003; Xu and He, 2020). Here, *C. lanceolata* accounted for over 90.55% of the total vegetation N storage and the soil (0–60 cm) stored an average of 87.64% of the total ecosystem stored N. When compared with the N stored in vegetation and the total amount of N fertilizer for 14 years, we found N loss was estimated to be over 86%. The N120 treatment showed the highest N recovery rate of all treatments, which did not support our second hypothesis. Although N recovery rate at extremely high doses of N fertilizer (240 kg N hm^−2^a^−1^) did not result in high N uptake, it is important to note the low N recovery rate (average 11.39%) and high loss of N (91.86% on average) after N fertilization, which indicate low plant N use efficiency. This is consistent with the hypothesis that increased atmospheric N deposition rates have led to lower plant N use efficiency (Liao et al., 2021).

Empirical evidence and meta-analysis indicate that N availability is a major factor determining plant growth (Kiba and Krapp, 2016; Liao et al., 2021). Plants can acquire N through their roots from soil inorganic (i.e., DON, NH_4_^+^-N and NO_3_^−^-N) and organic (e.g., urea and amino acids) compounds (Kiba and Krapp, 2016; Congreves et al., 2021). The balance of soil N nutrients affects the utilization of N (Aber et al., 1998; Nordin et al., 2001; Kiba and Krapp, 2016). For example, plant community-level N uptake rates can be affected by nitrogen availability (Dybzinski et al., 2021) and low N availability can increase plant N acquisition efficiency (Liao et al., 2021). Zhou et al. (2021) suggested that conifers use different soil N resources (i.e., nitrate and ammonium), and NO_3_^−^-N is a relatively large proportion (42%–52%) of total plant N uptake. Previous research from the same experimental site studied here showed that NH_4_^+^-N and NO_3_^−^-N concentration in *C. lanceolata* increase with N deposition rate after 12 years N fertilization, with stronger responses from NO_3_^−^-N (Shen et al., 2018), while medium-N and high-N significantly inhibited soil microbial biomass nitrogen with decreased percentage of 27.94%–29.50% (Shen et al., 2019b). However, there is not necessarily a significant association between N use efficiency and soil N availability, and soil P and plant P content can have strong effects on plant N use efficiency (Liao et al., 2021). We have an ongoing study to determine how changes in soil P and plant P content over time affect ecosystem N storage and N use efficiency after long-term N fertilization. The N recovery rate in this study is primarily based on natural N forms. The ^15^N tracing method provides a good approach to study the fate and retention of N inputs in forest ecosystems over long time scales (Brumme et al., 2009; Gurmesa et al., 2016; Congreves et al., 2021). For example, in an N saturated old-growth tropical forest, Gurmesa et al. (2016) used ^15^N tracer to estimate plant N recovery and total N retention in N-treatment plots.

Although we report several novel findings, there were limitations in our study design that could be addressed in future research. For example, calculating the N use efficiency without considering an increase or decrease in the soil N pool may lead to biased results (Tian et al., 2011). Vegetation as an important source of active N and changes in N storage in different vegetation types have high uncertainties (Xu et al., 2020). We focused only on *C. lanceolata* plantations, and studies of different tree species are needed to understand the generality of our findings, because plant community composition plays a key role in the use of different N sources (Nordin et al., 2001; Torre and Ávila, 2021). In addition, seasonal N cycling is important to forest N use (Li and Coleman, 2019); more vegetation samples should be collected during different growth seasons and parts (current-year, previous-year, or 2-year-old growth) to increase representation and generalization (Congreves et al., 2021). What’s more, detrital biomass, mainly from coarse woody debris, is a large N sink in forests (Wu et al., 2020), and it is necessary to determine how the mass loss from detritus biomass influences ecosystem N pools. Therefore, to assess the applicability of our findings beyond subtropical conifer plantation management, additional studies are needed covering a broad range of species, their N storage at different rates, and duration of N fertilization.

## Conclusion

Our study highlights the importance of considering various plant organs and ecosystem components in accurately estimating ecosystem N storage. Our results partially support our first hypothesis—that N storage would increase with N fertilization—but do not support our second hypothesis, that N recovery rate would decrease with N fertilization. With increasing N application rate, *C. lanceolata* N storage increased but ecosystem N storage did not increase beyond a threshold of medium-N fertilization after 14 years. The total ecosystem N storage in fertilized plots was 1.1–1.4 times higher than that of control plots. Taken together, our results suggest that long-term N fertilization lowered N recovery rate and N use efficiency. These findings underscore the progress that has been made towards assessing N storage and regional N cycling.

## Data Availability Statement

The raw data supporting the conclusions of this article will be made available by the authors, without undue reservation.

## Author Contributions

FS contributed to data curation, formal analysis, writing—original draft, visualization, conceptualization, and investigation. WL contributed to supervision, conceptualization, methodology, and resources. HD contributed to conceptualization, methodology, and writing—review and editing. JW contributed to supervision, methodology, project administration, software, and validation. CW contributed to writing—review and editing and investigation. YL contributed to writing—review and editing, validation, and funding acquisition. YY contributed to methodology, software, writing—review and editing, and funding acquisition. HF contributed to supervision, project administration, and funding acquisition. All authors contributed to the article and approved the submitted version.

## Funding

This study was financially supported by the National Natural Science Foundation of China (grants nos. 31960308, 31760167, 31971497, and 32160361), the Xingdian Scholar Fund of Yunnan Province, and the Double Top University Fund of Yunnan University. We also received support from the Key Laboratory of Soil Erosion and Control of Jiangxi Province, 2020 Open Research Fund Project.

## Conflict of Interest

The authors declare that the research was conducted in the absence of any commercial or financial relationships that could be construed as a potential conflict of interest.

## Publisher’s Note

All claims expressed in this article are solely those of the authors and do not necessarily represent those of their affiliated organizations, or those of the publisher, the editors and the reviewers. Any product that may be evaluated in this article, or claim that may be made by its manufacturer, is not guaranteed or endorsed by the publisher.

## Supplementary Material

Supplementary Figure 1 Mean annual precipitation and mean annual temperature of the study site (climatology based on measurements over 14 years from 2004 to 2017).

Supplementary Figure 2 Average DBH per treatment after 14 years N fertilization. N0, control; N60, low-N; N120, medium-N; N240, high-N. The values show means ± SE (*n* = 3).

Supplementary Figure 3 Basal area increment **(A)** and annual basal area increment **(B)** from 1992-2017 under different N treatments. N0, control; N60, low-N; N120, medium-N; N240, high-N. The values show means ± SE (*n* = 3). “*” indicated differences at P<0.05 between different treatments.

Supplementary Figure 4 Changes of understory layer species diversity **(A)** and biomass **(B)** under different N fertilization. N0, control; N60, low-N; N120, medium-N; N240, high-N. The values show means ± SE (*n* = 3). Lowercase letters indicate significant differences at *p* < 0.05 between different treatments.

Supplementary Figure 5 Total litter input biomass **(A)** and leaf and branch biomass in L and F layers **(B)** under different N treatments. L-leaf: litter leaf of undecomposed layer; L-branch: litter branch of undecomposed layer; F-leaf: litter leaf of semi-decomposed layer; F-branch: branch of semi-decomposed layer. N0, control; N60, low-N; N120, medium-N; N240, high-N. The values show means ± SE (*n* = 3). Lowercase letters indicate significant differences at *p* < 0.05 between different treatments.
