# Supplementary figures and images for: High N Storage but Low N Recovery After Long-Term N-Fertilization in a Subtropical Cunninghamia lanceolata Plantation Ecosystem: A 14-Year Case Study
Source: Front Plant Sci. 2022 Jun 15;13:914176. doi: 10.3389/fpls.2022.914176 (PMC9255632; doi:10.3389/fpls.2022.914176)

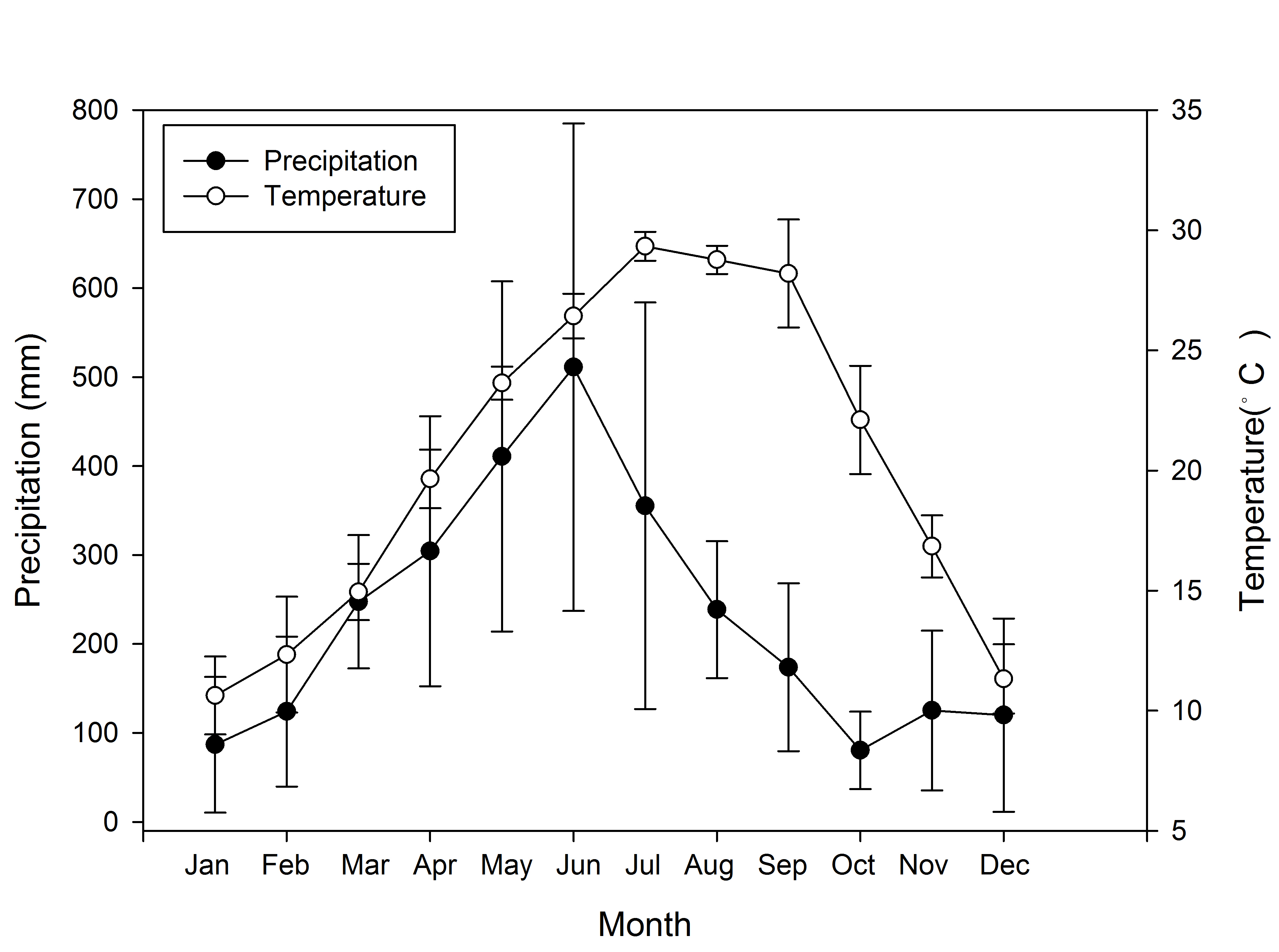

Supplement: Supplementary Figure 1 — Mean annual precipitation and mean annual temperature of the study site (climatology based on measurements over 14 years from 2004 to 2017). [file Image_1.JPEG]

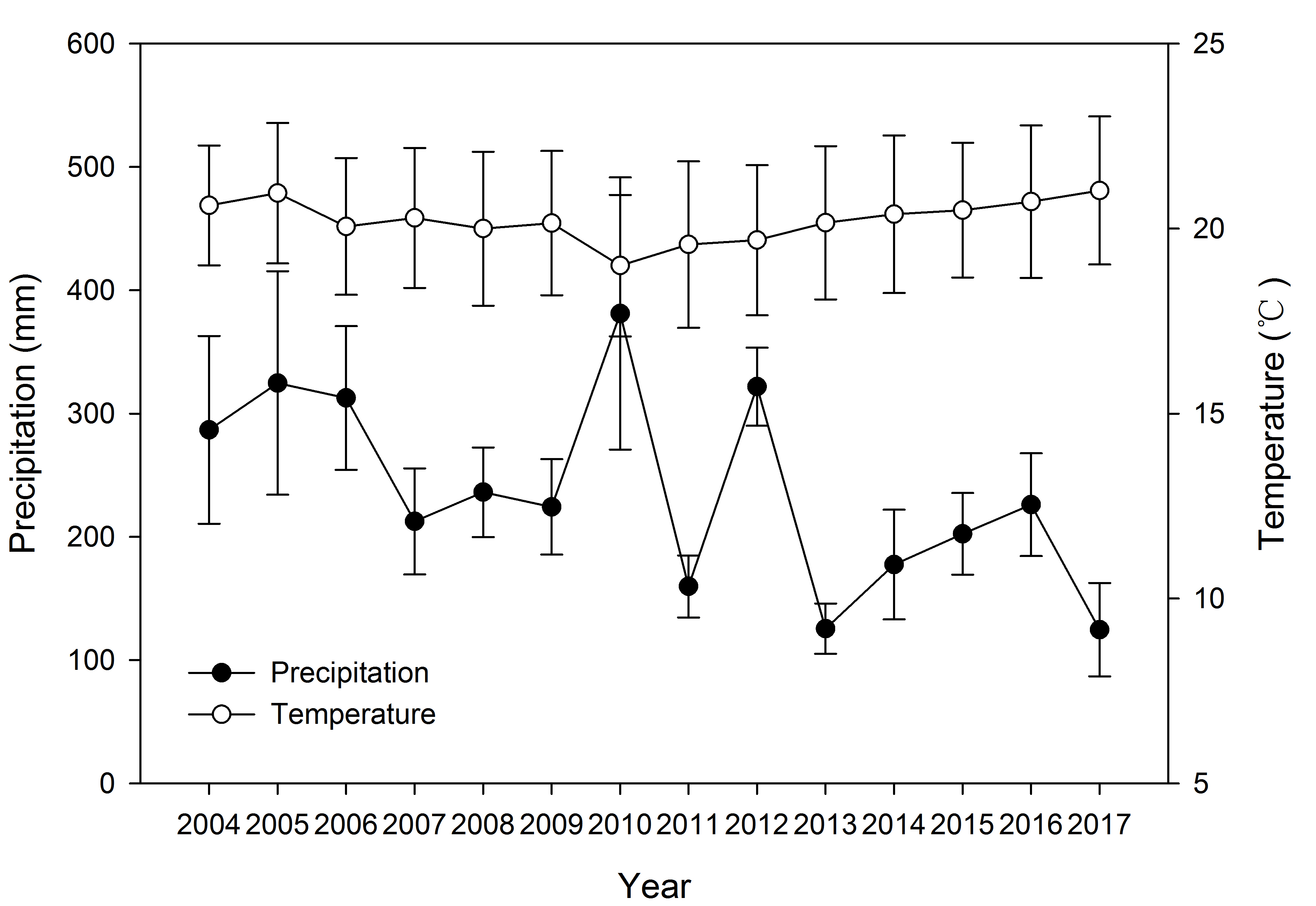

Supplement: Supplementary Figure 2 — Average DBH per treatment after 14 years N fertilization. N0, control; N60, low-N; N120, medium-N; N240, high-N. The values show means ± SE (n = 3). [file Image_2.JPEG]

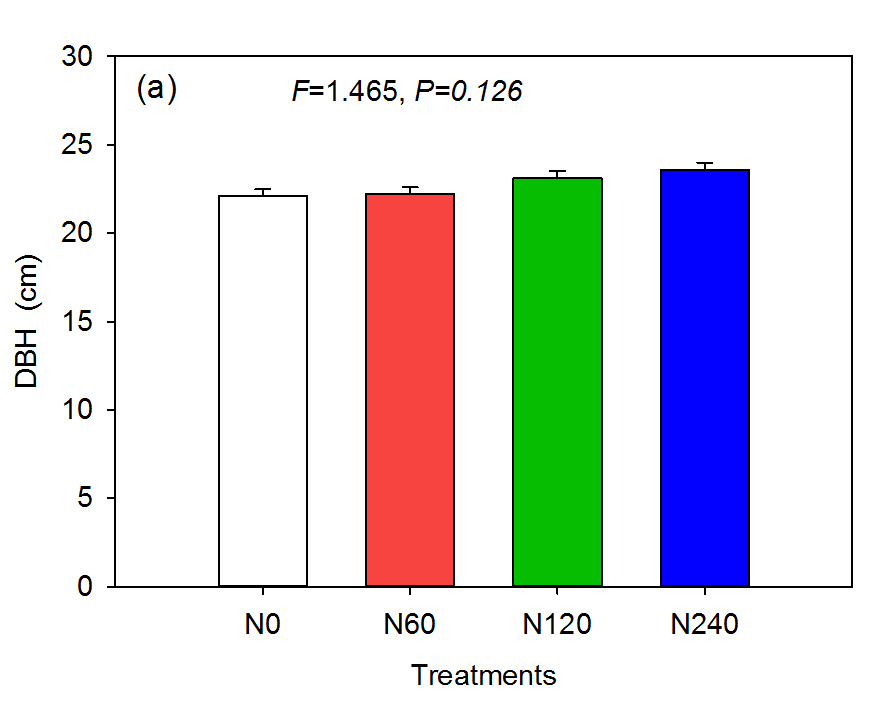

Supplement: Supplementary Figure 3 — Basal area increment (A) and annual basal area increment (B) from 1992-2017 under different N treatments. N0, control; N60, low-N; N120, medium-N; N240, high-N. The values show means ± SE (n = 3). “*” indicated differences at P<0.05 between different treatments. [file Image_3.JPEG]

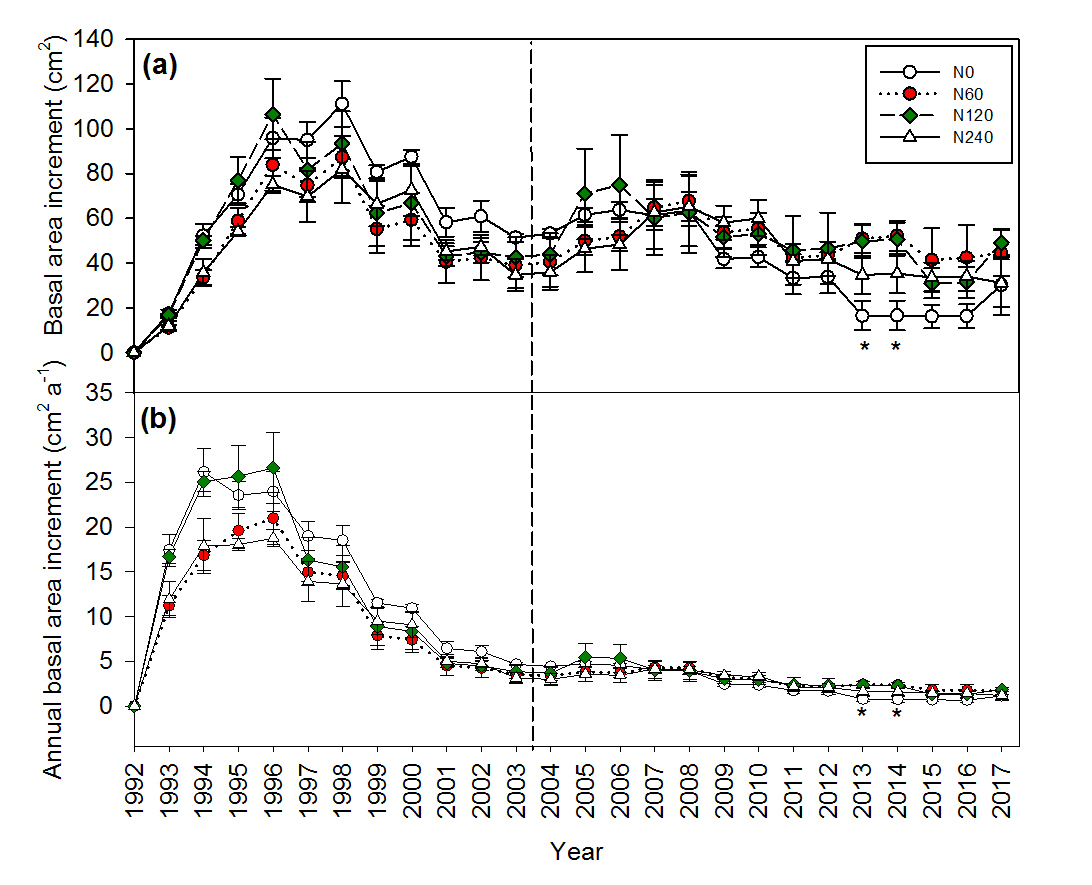

Supplement: Supplementary Figure 4 — Changes of understory layer species diversity (A) and biomass (B) under different N fertilization. N0, control; N60, low-N; N120, medium-N; N240, high-N. The values show means ± SE (n = 3). Lowercase letters indicate significant differences at p < 0.05 between different treatments. [file Image_4.JPEG]

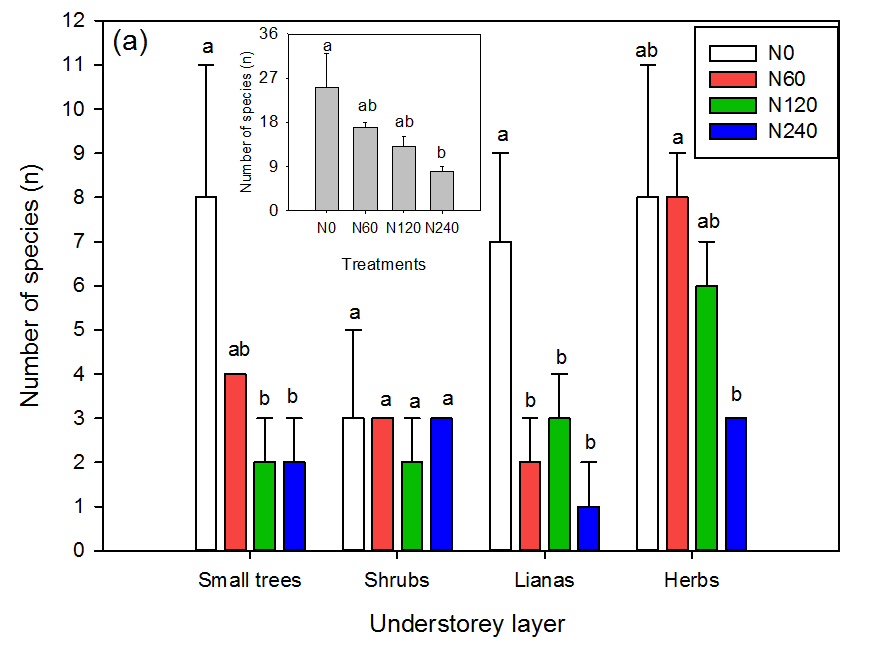

Supplement: Supplementary Figure 5 — Total litter input biomass (A) and leaf and branch biomass in L and F layers (B) under different N treatments. L-leaf: litter leaf of undecomposed layer; L-branch: litter branch of undecomposed layer; F-leaf: litter leaf of semi-decomposed layer; F-branch: branch of semi-decomposed layer. N0, control; N60, low-N; N120, medium-N; N240, high-N. The values show means ± SE (n = 3). Lowercase letters indicate significant differences at p < 0.05 between different treatments. [file Image_5.JPEG]

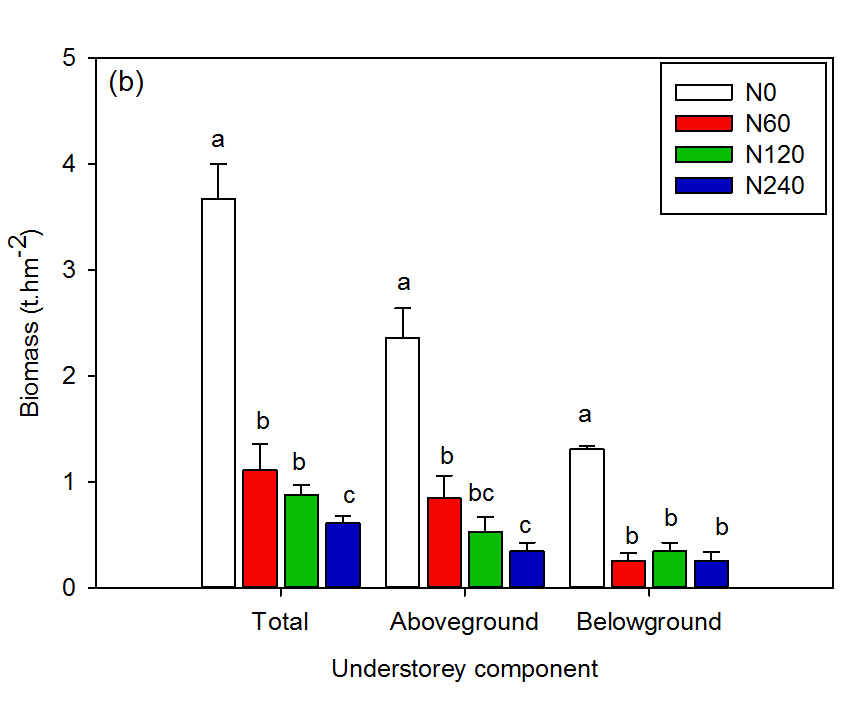

Supplement: Supplementary file 6 [file Image_6.JPEG]

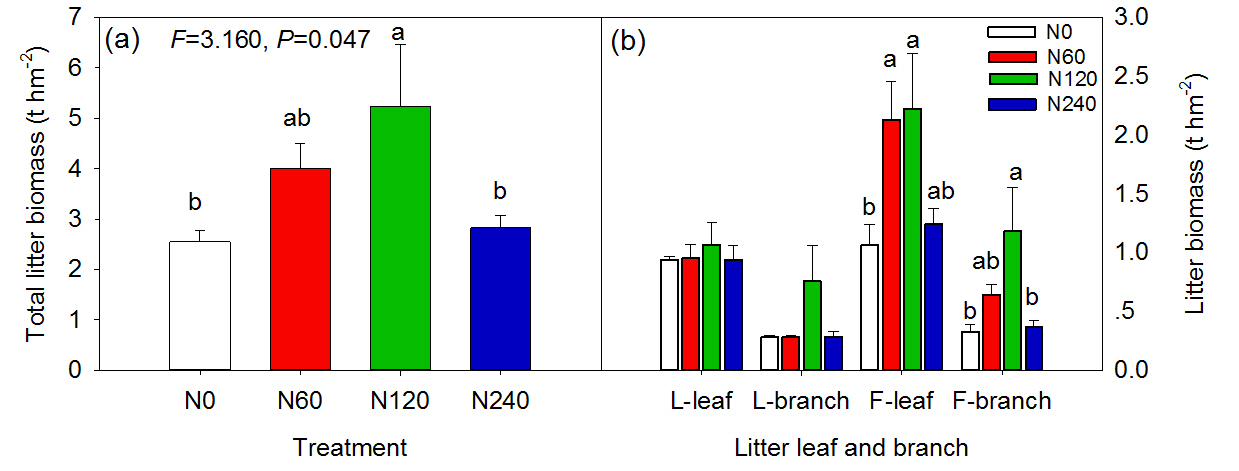

Supplement: Supplementary file 7 [file Image_7.JPEG]
