# Supplementary material for: High N Storage but Low N Recovery After Long-Term N-Fertilization in a Subtropical Cunninghamia lanceolata Plantation Ecosystem: A 14-Year Case Study
Source: Front Plant Sci. 2022 Jun 15;13:914176. doi: 10.3389/fpls.2022.914176 (PMC9255632; doi:10.3389/fpls.2022.914176)
Supplement: Supplementary file 8 [file Table_1.docx]

Supplementary table 1

**SUPPLEMENTARY TABLE 1丨**Dry mass and N content of Chinese fir plant organs under different N fertilizer treatments in 2003. Values are means ± SEs (n=3).

| **Component** | **Dry mass ( t hm^-2^)** | | | |  | **N content (g kg^-1^)** | | | |
| --- | --- | --- | --- | --- | --- | --- | --- | --- | --- |
|  | **N0** | **N60** | **N120** | **N240** |  | **N0** | **N60** | **N120** | **N240** |
| Leaves | 12.28 | 14.02 | 13.52 | 11.96 |  | 14.10 | 15.30 | 14.40 | 14.50 |
| Branches | 19.57 | 11.92 | 14.68 | 18.21 |  | 5.09 | 5.22 | 6.55 | 5.68 |
| Stems | 81.93 | 84.95 | 86.82 | 77.30 |  | 2.62 | 2.42 | 2.83 | 2.28 |
| Bark | 9.13 | 9.63 | 10.00 | 8.95 |  | 6.97 | 5.27 | 7.00 | 7.13 |
| Coarse roots | 21.25 | 25.81 | 22.21 | 19.28 |  | 0.65 | 0.72 | 0.74 | 0.76 |
| 2-5 mm | 0.27 | 0.31 | 0.53 | 0.27 |  | 0.71 | 0.72 | 0.72 | 0.79 |
| <2 mm | 0.18 | 0.26 | 0.30 | 0.17 |  | 0.68 | 0.70 | 0.74 | 0.76 |
| Subtotal | 144.61 | 146.91 | 148.07 | 136.13 |  |  |  |  |  |
