# Supplementary material for: High N Storage but Low N Recovery After Long-Term N-Fertilization in a Subtropical Cunninghamia lanceolata Plantation Ecosystem: A 14-Year Case Study
Source: Front Plant Sci. 2022 Jun 15;13:914176. doi: 10.3389/fpls.2022.914176 (PMC9255632; doi:10.3389/fpls.2022.914176)
Supplement: Supplementary file 9 [file Table_2.docx]

Supplementary table 2

**SUPPLEMENTARY TABLE 2** **丨**Loss of N by vegetation after 14 years N fertilization at the rates of 60, 120, and 240 kg N hm^-2^a^-1^ relative to the control

| **N fertilizer rate**  **(kg N hm^-2^a^-1^)** | **N storage in vegetation**  **(kg N hm^-2^)** | **Total amount of N fertilizer**  **in 14 years (kg N hm^-2^)** | **Loss of N**  **(kg N hm^-2^)** |
| --- | --- | --- | --- |
| N60 | 14.44 | 840 | 825.56 |
| N120 | 229.27 | 1680 | 1450.73 |
| N240 | 304.07 | 3360 | 3055.93 |
